# Selected Clostridia Strains from The Human Microbiota and their Metabolite, Butyrate, Improve Experimental Autoimmune Encephalomyelitis

**DOI:** 10.1007/s13311-021-01016-7

**Published:** 2021-04-07

**Authors:** Laura Calvo-Barreiro, Herena Eixarch, Thais Cornejo, Carme Costa, Mireia Castillo, Leyre Mestre, Carmen Guaza, María del Carmen Martínez-Cuesta, Takeshi Tanoue, Kenya Honda, Juan José González-López, Xavier Montalban, Carmen Espejo

**Affiliations:** 1 Servei de Neurologia-Neuroimmunologia, Vall d’Hebron Institut de Recerca, Hospital Universitari Vall d’Hebron, Centre d’Esclerosi Múltiple de Catalunya, Pg. Vall d’Hebron 119-129, 08035 Barcelona, Spain; 2 Universitat Autònoma de Barcelona, 08193 Bellaterra Cerdanyola del Vallès, Spain; 3 Red Española de Esclerosis Múltiple (REEM), Instituto de Salud Carlos III, Ministerio de Economía y Competitividad, Fondo de Investigación Sanitaria, Madrid, Spain; 4 Servei de Microbiologia, Hospital Universitari Vall d’Hebron, Pg. Vall d’Hebron 119-129, 08035 Barcelona, Spain; 5 Grupo de Neuroinmunología, Departamento de Neurobiología Funcional y de Sistemas, Instituto Cajal, CSIC, Avda. Doctor Arce 37, 28002 Madrid, Spain; 6 Department of Food Biotechnology and Microbiology, Instituto de Investigación en Ciencias de la Alimentación, CIAL (CSIC-UAM), C/ Nicolás Cabrera 9, 28049 Madrid, Spain; 7 RIKEN Center for Integrative Medical Sciences, 1-7-22 Suehiro-cho, Tsurumi-ku, Yokohama, Kanagawa 230-0045 Japan

**Keywords:** Gut Microbiota, Clostridia Strains, Short-chain Fatty Acid, Immune Regulation, Experimental Autoimmune Encephalomyelitis, Multiple sclerosis

## Abstract

Gut microbiome studies in multiple sclerosis (MS) patients are unravelling some consistent but modest patterns of gut dysbiosis. Among these, a significant decrease of Clostridia cluster IV and XIVa has been reported. In the present study, we investigated the therapeutic effect of a previously selected mixture of human gut-derived 17 Clostridia strains, which belong to Clostridia clusters IV, XIVa, and XVIII, on the clinical outcome of experimental autoimmune encephalomyelitis (EAE). The observed clinical improvement was related to lower demyelination and astrocyte reactivity as well as a tendency to lower microglia reactivity/infiltrating macrophages and axonal damage in the central nervous system (CNS), and to an enhanced immunoregulatory response of regulatory T cells in the periphery. Transcriptome studies also highlighted increased antiinflammatory responses related to interferon beta in the periphery and lower immune responses in the CNS. Since Clostridia-treated mice were found to present higher levels of the immunomodulatory short-chain fatty acid (SCFA) butyrate in the serum, we studied if this clinical effect could be reproduced by butyrate administration alone. Further EAE experiments proved its preventive but slight therapeutic impact on CNS autoimmunity. Thus, this smaller therapeutic effect highlighted that the Clostridia-induced clinical effect was not exclusively related to the SCFA and could not be reproduced by butyrate administration alone. Although it is still unknown if these Clostridia strains will have the same effect on MS patients, gut dysbiosis in MS patients could be partially rebalanced by these commensal bacteria and their immunoregulatory properties could have a beneficial effect on MS clinical course.

**Supplementary Information:**

The online version contains supplementary material available at 10.1007/s13311-021-01016-7.

## Introduction

Multiple sclerosis (MS) is an autoimmune, degenerative, chronic, and demyelinating disease that affects the central nervous system (CNS) [1]. As a complex disease, MS depends on both genetic and environmental factors and it is thought to be developed in genetically predisposed individuals after exposure to different environmental factors. Ground breaking data from research in experimental autoimmune encephalomyelitis (EAE) models and subsequent studies in MS patients have connected gut microbiota to innate and adaptive immune responses characteristic of MS pathogenesis [2].

Gut microbiome studies in MS patients are unravelling some consistent but modest patterns of gut dysbiosis compared to healthy controls [3–9]. Among these gut microorganisms associated to MS disease course, a significant decrease of Clostridia cluster IV and XIVa has been reported [5]. On the other hand, previous *in vivo* studies have shown that a cocktail of 17 Clostridia strains isolated from a human faecal sample, which partially belong to previously-mentioned clusters, promoted both the number and function of regulatory T (Treg) cells in the colon and successfully suppressed experimental colitis in mice [10]. Subsequent analysis of caecal contents from Clostridia-treated mice showed a higher concentration of short-chain fatty acids (SCFAs): acetate, propionate, butyrate, and isobutyrate [10]. Simultaneously, it was demonstrated that both propionate and butyrate promoted the generation of splenic Treg cells *in vivo* [11]. Similarly, naïve CD4^+^ T cells treated with propionate and butyrate *in vitro* promoted Treg cell populations and, more important, a propionate-rich diet ameliorated the clinical course of EAE mice due to an increase in Treg cell population and interleukin (IL)-10 production [12]. Keeping this in mind, it is reasonable that the reduction of these Clostridia clusters may be associated with the pathogenesis of MS due to their intrinsic immunoregulatory properties depending, to a greater or lesser extent, on SCFA production.

In the present study, we investigated the therapeutic effect of a previously selected mixture of 17 Clostridia strains from the human microbiota on the clinical outcome of established EAE. We observed a clinical improvement that was related to lower demyelination and astrocyte reactivity as well as a trend to lower microglia reactivity/infiltrating macrophages and axonal damage in the CNS, and to an enhanced immunoregulatory response of Treg cells in the periphery. Likewise, microarrays studies highlighted increased antiinflammatory responses related to interferon beta (IFN-β) production in the periphery and lower activation, differentiation, and proliferation of immune cells in the CNS. Finally, higher levels of the immunoregulatory SCFA butyrate were found in the serum of Clostridia-treated mice. Further *in vivo* assays with the SCFA butyrate proved its preventive effect on CNS autoimmunity but a slight therapeutic clinical impact on EAE clinical course. Our results suggest that the therapeutic effect performed by this commensal bacteria population was not exclusively related to the SCFA and could not be reproduced by butyrate administration alone. Although it is still unknown whether these 17 Clostridia strains will have the same effect on MS patients, previously-defined gut dysbiosis regarding Clostridia cluster IV and XIVa could be compensated by bacteria administration and their immunoregulatory properties could have a beneficial effect on MS clinical course.

## Methods

### Mice

C57BL/6JOlaHsd 8-week-old female mice purchased from Envigo (Venray, Holland) were used. Mice were housed under standard light- and climate-controlled conditions, and standard chow and water were provided ad libitum. All experiments were performed in strict accordance with EU (Directive 2010/63/EU) and Spanish regulations (Real Decreto 53/2013; Generalitat de Catalunya Decret 214/97). The Ethics Committee on Animal Experimentation of the Vall d'Hebron Research Institute approved all procedures described in this study (protocol number: 35/15 CEEA).

### Induction and Assessment of EAE

Anaesthetised mice were immunised by subcutaneous injection of 100 μl of phosphate-buffered saline containing 200 μg of peptide 35–55 from myelin oligodendrocyte glycoprotein (MOG_35–55_, Proteomics Section, Universitat Pompeu Fabra, Barcelona, Spain) emulsified in 100 μl of complete Freund’s adjuvant [incomplete Freund’s adjuvant (IFA, F5506, Sigma-Aldrich, St. Louis, MO, USA) containing 4 mg/ml *Mycobacterium tuberculosis* H37RA (231141, BD Biosciences, BD, Franklin Lakes, NJ, USA)]. At 0 and 2 days postimmunisation (dpi), mice were intravenously injected with 250 ng of *pertussis toxin* (P7208, Sigma-Aldrich). Mice were weighed and examined daily for neurological signs in a blinded manner and corrective measures and endpoint criteria to ensure EAE-incident animals welfare were included as previously detailed [13]. All data presented are in accordance with the ARRIVE guidelines for animal research and with the guidelines suggested for EAE publications [14].

### Motor Function Assessment

The day before the end of the experiment (27 or 34 dpi), motor performance was evaluated using a Rotarod apparatus (Ugo Basile, Gemonio, Italy) that was set to accelerate from a speed of 4 to 40 rotations per minute in a 300-s time trial [13]. Briefly, once mice were placed on the rotating cylinder, the amount of time (sec) that the animals walked on the cylinder without falling was recorded. Each mouse was given four trials, and the value for each mouse depicts the mean of these four measurements.

### Bacterial Strains and Treatments

A consortium of 17 Clostridia strains was originally isolated from a stool sample of a Japanese healthy volunteer as described previously [10]. The bacterial isolates were individually cultured in BD Schaedler Agar with Vitamin K1 and 5% Sheep Blood (254042, BD) in an anaerobic jar (Biomérieux, Marcy l’Etoile, France) under strict anaerobic conditions at 37ºC during three to four days. To prepare the bacterial mixture, Clostridia strains were again individually grown in BD BBL Thioglycollate Medium (221742, BD) in an anaerobic jar under strict anaerobic conditions at 37ºC until they reached confluence. Later, they were mixed at equal amounts of media volume in an anaerobic chamber and stored in ready-to-use individual cryotubes supplemented with 5% glycerol at -80ºC until use.

Preventive approach: Since oral gavage administration diminished EAE incidence in a pilot study as a preventive approach (*data not shown*), the butyrate treatment was administered via water feeding bottle. Seven days before mice immunisation, a 50 ml water volume containing 1.1 g of sodium butyrate (200 mM, 303410, Sigma-Aldrich) or not supplemented (vehicle) was prepared daily and added in the water feeding bottle of the corresponding cage. This concentration of butyrate was selected taking into account previous research on SCFA propionate in the EAE model [12]. The following day, the remaining volume was measured and the intake of sodium butyrate per mouse was estimated daily. Daily treatment was performed until the end of the experiment (35 dpi).

Therapeutic approach: After attaining a clinical score equal to or greater than 1 (between 12 and 15 dpi), mice were randomised into clinically equivalent experimental groups. Administration of a 250 µl volume containing Clostridia strains or Thioglycollate Medium, supplemented with 5% glycerol (vehicle) via oral gavage was performed once daily until the end of the experiment (28 dpi). Regarding butyrate treatment, after attaining a clinical score equal to or greater than 1 (between 13 and 15 dpi), mice were randomised into clinically equivalent experimental groups. Administration of a 200 µl volume containing 100 mg of sodium butyrate or water (vehicle) via oral gavage was performed once daily until the end of the experiment (28 dpi).

### *Ex vivo* Splenocyte Proliferative Capacity

Splenocyte suspensions were prepared by grinding spleens through a 70-μm nylon cell strainer at the end of the experiment (28 or 35 dpi). Splenocytes were seeded at 2 × 10^5^ cells per well in supplemented X-VIVO™ 15 medium (BE02-060F, Lonza, Basel, Switzerland) within 96-well plates as previously detailed [13]. Splenocyte cultures were stimulated with 5 μg/ml MOG_35-55_ (antigen-specific stimulus) or 5 μg/ml phytohaemagglutinin-L (PHA-L, L2769, Sigma-Aldrich) (polyclonal stimulus) and compared to non-stimulated (control) condition. After 54 h, 75 μl of supernatant were harvested and stored at − 80 °C to further assess cytokine secretion. At the same time, 75 μl of supplemented medium containing 1 μCi of [^3^H]-thymidine (NET027Z, PerkinElmer, Waltham, MA, USA) were added to each well. Splenocyte cultures were maintained under the same conditions for an additional 18 h and incorporated radioactivity was measured in a beta-scintillation counter (Wallac, Turku, Finland). Five replicates per experimental condition (MOG_35-55_, PHA-L, and control) were assessed per mouse. Stimulation indices were calculated by dividing the mean counts per minute (cpm) of MOG_35-55_ or PHA-L condition by the mean cpm of the control condition. The results are shown as the stimulation index.

### Cytokine Detection by Luminex

Cytokine secretion pattern [granulocyte–macrophage colony-stimulating factor (GM-CSF), IFN-γ, IL-2, IL-4, IL-6, IL-10, IL-12p70, IL-17A, IL-21, IL-22, IL-23 and tumour necrosis factor alpha (TNF-α)] was assessed in the supernatants of MOG_35-55_-stimulated splenocytes by using a ProcartaPlex Multiplex Immunoassay (Invitrogen, Carlsbad, CA, USA), according to the manufacturer’s instructions. Samples were acquired in a Magpix instrument (Luminex Corporation, Austin, TX, USA) and data were analysed with a ProcartaPlex Analyst software (Thermo Fisher Scientific, Waltham, MA, USA). The results are shown as the concentration of each cytokine (pg/ml).

### Flow Cytometry Analysis

Spleen cell suspensions were prepared as described previously. Cell subsets were analysed using fluorochrome-conjugated monoclonal antibodies (mAbs) after discrimination of dead cells by Fixable Viability Stain (BD Pharmingen). For analysis of the Treg cell population, CD3ε (BD Biosciences Cat# 553,061, RRID: AB_394594), CD4 (BD Biosciences Cat# 561,090, RRID:AB_10562560), and CD25 (Thermo Fisher Scientific Cat# 12–0251-81, RRID:AB_465606) were used. FoxP3 intracellular staining was performed using fluorochrome-labelled anti-FoxP3 mAb (Thermo Fisher Scientific Cat# 17–5773-80, RRID:AB_469456). CD39 (Thermo Fisher Scientific Cat# 25–0391-80, RRID:AB_1210767), CD62L (BD Biosciences Cat# 563,252, RRID:AB_2738098), HELIOS (BD Biosciences Cat# 563,801, RRID:AB_2738428), and ICOS (BD Biosciences Cat# 564,592, RRID:AB_2738858) were also selected and evaluated in the Treg cell population. For dendritic cell subpopulations, mAbs specific for B220 (BD Biosciences Cat# 553,088, RRID:AB_394618), CD11b (BD Biosciences Cat# 562,605, RRID:AB_11152949), CD11c (BD Biosciences Cat# 553,802, RRID:AB_395061), CD8a (Thermo Fisher Scientific Cat# 47–0081-80, RRID:AB_1272221), and CD317 (Thermo Fisher Scientific Cat# 17–3172-82, RRID:AB_10596356) were used. For analysis of macrophage, neutrophils, and MDSC populations, CD11b (BD Biosciences Cat# 562,605, RRID:AB_11152949), CD206 (BioLegend Cat# 141,712, RRID:AB_10900420), F4/80 (Thermo Fisher Scientific Cat# 12–4801-82, RRID:AB_465923), Ly6C (BD Biosciences Cat# 560,593, RRID:AB_1727557), and Ly6G (BD Biosciences Cat# 551,460, RRID:AB_394207) were used; for B cell subsets, B220 (BD Biosciences Cat# 552,772, RRID:AB_394458), CD1d (BD Biosciences Cat# 553,846, RRID:AB_2073521), CD5 (BD Biosciences Cat# 550,035, RRID:AB_398457), CD19 (BD Biosciences Cat# 560,143, RRID:AB_1645234), CD138 (BD Biosciences Cat# 562,610, RRID:AB_11153126), and MHCII (BD Biosciences Cat# 562,363, RRID:AB_11153297) were used. For analysis of T cell activation status, CD3ε (BD Biosciences Cat# 553,062, RRID:AB_394595), CD4 (Thermo Fisher Scientific Cat# 46–0042-80, RRID:AB_1834432), CD8a (BD Biosciences Cat# 563,152, RRID:AB_2738030), CTLA-4 (BD Biosciences Cat# 564,331, RRID:AB_2738751), PD-1 (BioLegend Cat# 135,217, RRID:AB_10900085), and TIM-3 (Thermo Fisher Scientific Cat# 25–5870-82, RRID:AB_2573483) were used. For intracellular cytokine determination, *ex vivo* stimulation of splenocytes was performed as previously detailed [13]. Then, CD3ε (BD Biosciences Cat# 553,061, RRID:AB_394594), CD4 (Thermo Fisher Scientific Cat# 46–0042-80, RRID:AB_1834432), and CD8a (BD Biosciences Cat# 563,152, RRID:AB_2738030) staining was performed. Cytokine intracellular staining was performed using fluorochrome-labelled anti-IFN-γ (BD Biosciences Cat# 563,376, RRID:AB_2738165), anti-IL-4 (BD Biosciences Cat# 554,436, RRID:AB_398556), anti-IL-10 (BD Biosciences Cat# 563,276, RRID:AB_2738111), and anti-IL-17A (BD Biosciences Cat# 561,020, RRID:AB_10584331) mAbs, and anti-CD69 mAb (BD Biosciences Cat# 560,689, RRID:AB_1727506) to assess proper *ex vivo* stimulation. Samples were acquired in a CytoFLEX flow cytometer (Beckman Coulter, Brea, CA, USA) and data were analysed with CytExpert 2.3 software (Beckman Coulter).

### Histological Analysis

Preventive approach: At 35 dpi, spinal cords of euthanised mice were collected, fixed in a 4% paraformaldehyde solution, embedded in paraffin, and cut into 4-μm thick coronal sections. Demyelination was assessed by Klüver-Barrera staining and inflammatory infiltration by haematoxylin and eosin staining, as previously described [15]. Images were acquired using a Leica DFC550 fluorescence microscope (Leica Microsystems, Wetzlar, Germany) and LAS V4.5 visualisation software (Leica Microsystems), obtained at a magnification of 10X, and analyzed using ImageJ software. For every staining, one mosaic image from the thoracic spinal cord was selected per mouse and evaluated in a blinded manner. The results are shown as the individual score per mouse.

Therapeutic approach: At 28 dpi, spinal cords of euthanised mice were collected, fixed in a 4% paraformaldehyde solution, embedded in paraffin, and cut into 4-μm thick coronal sections. Demyelination was assessed by rabbit polyclonal anti-myelin basic protein (MBP, Millipore Cat# AB980, RRID:AB_92396); T cells, by anti-CD3 (Agilent Cat# A0452, RRID:AB_2335677); axonal damage, by mouse monoclonal purified anti-neurofilament H, nonphosphorylated antibody (SMI32, BioLegend Cat# 801,701, RRID:AB_2564642); and reactive microglia/infiltrating macrophages and astroglia reactivity, by lectin from *Lycopersicon esculentum* (LEA, L0401, Sigma-Aldrich) and mouse monoclonal anti-glial fibrillary acidic protein (GFAP, Sigma-Aldrich Cat# C9205, RRID:AB_476889), respectively. Immunofluorescences were carried out as previously described [13]. Images were acquired using a Leica AF6000 fluorescence microscope (Leica Microsystems) and LAS AF visualisation software (Leica Microsystems), and mosaic images were obtained at a magnification of 20X and analysed using ImageJ software as previously detailed [13].

### *In vivo* Intestinal Permeability

At 28 dpi, EAE mice were weighed and orally gavaged with an isotonic solution of 0·9% NaCl with fluorescein sodium salt (NaF, F6377, Sigma-Aldrich) at 10 μg/g mouse body weight or without NaF (negative control mice). After 1 h, mice were euthanised, blood samples were collected in heparinised tubes, and NaF concentration in plasma was measured by spectrophotofluorimetry in a Thermo Scientific Appliskan (Thermo Fisher Scientific) as previously described [13, 16]. NaF standard concentrations were used as reference and both samples and calibration curve were performed in duplicate.

### Transcriptome Studies

At 28 dpi, spinal cords of euthanised mice were collected, immersed in liquid nitrogen, and stored at -80ºC until use. Total RNA was isolated from spinal cords using QIAzol lysis reagent (79306, Qiagen, Hilden, Germany) and RNeasy Mini Kit (74104, Qiagen) with on-column DNase digestion (79254, Qiagen) in order to remove any genomic DNA trace according to the manufacturer's instructions. Regarding splenocytes, splenocyte suspensions were prepared as described previously at the end of the experiment (28 dpi), aliquoted in fetal bovine serum (FBS, Biowest, Nuaillé, France) supplemented with 10% dimethyl sulfoxide (DMSO), and stored in liquid nitrogen until use. RNeasy Mini Kit together with on-column DNase digestion was also used to isolate splenocyte total RNA according to the manufacturer's instructions. Prior to cDNA generation with WT Pico Reagent Kit-HT (Applied Biosystems, Thermo Fisher Scientific), RNA quality was analysed by capillary electrophoresis (Bioanalyzer 2100, Agilent, Santa Clara, CA, USA). Subsequently, Clariom S Pico Assay HT, mouse arrays were processed according to the manufacturer’s instructions in a GeneTitan Multi-Channel instrument (Applied Biosystems, Thermo Fisher Scientific).

### Determination of SCFA Levels in Serum

Mice were euthanised and the total blood volume was collected by cardiac puncture in non-heparinised tubes at the end of the experiment (28 dpi). Blood samples were kept on ice until further processing. Next, blood samples were centrifuged at 3,500 revolutions per minute (rpm) and 4ºC for 15 min to separate the serum. Finally, serum samples were obtained and stored at -80ºC until use. Approximately 200 µl of accurately measured serum samples were individually mixed with 30 µl of the internal standard (consisting in 100 µg/mL of 4-methylvaleric acid in methanol) and 300 µl of acetonitrile. After protein precipitation, the mixtures were centrifuged at 5,000 rpm and the supernatant transferred to a clean microtube and derivatised with *o*-benzyl hydroxylamine as previously reported [17]. The derivatised analytes were detected by a selected reaction monitoring (SRM) method. Briefly, 50 µl of *N*-(3-dimethylaminopropyl)-*N'*-ethylcarbodiimide hydrochloride 1 M were mixed with 50 µl of *o*-benzyl hydroxylamine 1 M and added into the mixture. Extracts were left at room temperature and shaken every 15 min in a vortex. After one hour, 1 ml of ultrapure water was added. Derivatised extracts were transferred to a clean 10 ml tube and mixed with 4 ml of ethyl acetate. After vigorous shaking for one minute, samples were centrifuged at 3,800 rpm for 5 min and the organic layer was separated and dried under nitrogen stream. The extracts were reconstituted in 1 ml water:methanol (50:50) and 3 µl were injected into the liquid chromatography tandem mass spectrometry (LC–MS/MS) system. The LC–MS/MS system consisted in an Acquity ultra-performance liquid chromatography system (Waters, Milford, MA, USA) coupled to a triple quadrupole mass spectrometer provided with an electrospray interface (Xevo TQ-S micro, Waters). The chromatographic separation was performed in an Acquity UPLC BEH C18 column (Waters). Concentrations of SCFA were obtained by external calibration. Four quality control samples, which consisted of water spiked with known amounts of the analytes, were injected in the same batch as the samples. Additionally, four blank samples were included in the batch to evaluate the ubiquitous contribution of each metabolite. Samples were processed using the TargetLynx software (Waters).

### Statistics

When two experimental groups were compared in an only independent experiment, all study variables were analysed using the Student t-test. When variances were equal or different between experimental groups, Satterthwaite or Pooled method was used, respectively. All analyses were carried out with the Proc TTEST procedure. All tests were two-tailed, and statistical significance was set at a *p value* of < 0·05. When two or more experimental groups were compared in several independent experiments, all study variables were analysed using the differences of least-squares means. Normal or lognormal distributions were assumed for all studied variables depending on data distribution. When repeated measures within mice were performed, compound symmetry was used as the covariance structure. When only a potential clustering effect of the experiments was present, a variance components structure showed an acceptable fit. All analyses were carried out with the Proc MIXED program, except for analysis of lognormal distribution variables, for which Proc GLIMMIX was used. All tests were two-tailed, and statistical significance was set at a *p value* of < 0·05.

For the transcriptome studies, the statistical analysis of the data was performed using the statistical language R (R version 3.6.1, Copyright© 2018 The R Project for Statistical Computing) and the libraries developed for the microarray analysis in the Bioconductor Project (https://www.bioconductor.org). The analysis of biological significance has been based on GSEA on different annotation databases [18]. The analysis has been performed over two annotation databases: the "Gene Ontology" (GO) and the Reactome Pathway Knowledge base [19].

### Data Sharing

Completed metadata worksheet, raw data, and processed data from the microarray studies were deposited in GEO database: GSE155438.

## Results

### Clostridia Administration Improves the EAE Clinical Outcome as a Therapeutic Approach

**Fig. 1:**
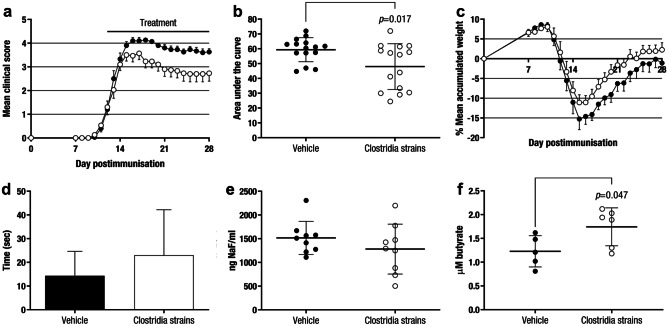
**Clostridia Strains Improve the Clinical Outcome of EAE Mice as a Therapeutic Approach.** C57BL/6JOlaHsd mice were immunised by subcutaneous injection of MOG_35–55_ and weighed and examined daily for neurological signs. After attaining a clinical score equal to or greater than 1 and being randomised (12–15 dpi), mice were treated once daily with Clostridia strains (250 μl confluent Clostridia culture) or vehicle (250 μl thioglycollate medium), weighed and examined daily for neurological signs in a blinded manner until the end of the experiment (28 dpi). Daily oral therapeutic treatment with Clostridia strains improves the clinical score **a **and the AUC **b** and does not affect weight loss **c** or motor coordination skills **d** compared to vehicle group. The charts present the combined results of two independent experiments (n = 15 mice per group). At 28 dpi, EAE animals were weighed and orally gavaged with an isotonic solution of NaF at 10 μg/g mouse body weight. After 1 h, mice were euthanised, and plasma samples were collected. Intestinal permeability was evaluated by measuring the NaF concentration in mouse plasma and no differences were observed between the experimental groups **e**. The graph presents the results of a representative experiment (n = 9 mice per group). Moreover, higher levels of the SCFA butyrate are presented in the serum of Clostridia-treated mice **f**. The graph presents the results of a representative experiment (n = 6 mice per group). The data are presented as the means ± standard errors of the mean **a**,** c **or the means ± standard deviations **b**, **d**, **e**, **f**. Black circle, Vehicle; white circle, Clostridia strains. Abbreviations: NaF: fluorescein sodium salt

Mice were treated once daily with Clostridia strains or vehicle after attaining a clinical score equal to or greater than 1 and being randomised into clinically equivalent experimental groups, from 12–15 dpi to the end of the experiment (28 dpi). Treatment with Clostridia strains improved the clinical outcome [area under the curve (AUC): 48·00 ± 15·41, n = 15, *p* = 0·017] compared to vehicle treatment (AUC: 59·38 ± 8·09, n = 15) (Fig. 1a,b). Mice were weighed daily to monitor their well-being and the Rotarod test was performed to assess motor coordination skills at 27 dpi. No statistically significant differences were observed regarding weight loss (*p* = 0·190) (Fig. 1c) or the Rotarod test (Vehicle: 14·37 ± 10·26 s, n = 15, vs. Clostridia strains: 23·03 ± 19·16 s, n = 15, *p* = 0·361) (Fig. 1d). Finally, no differences were observed regarding intestinal permeability in the Clostridia strains group (1281·66 ± 525·50 ng of NaF/ml, n = 9, *p* = 0·280) compared to the vehicle group (1516·88 ± 349·60 ng NaF/ml, n = 9) (Fig. 1e).

### Pathological Signs are Reduced in the CNS of Clostridia-Treated EAE Mice

**Fig. 2:**
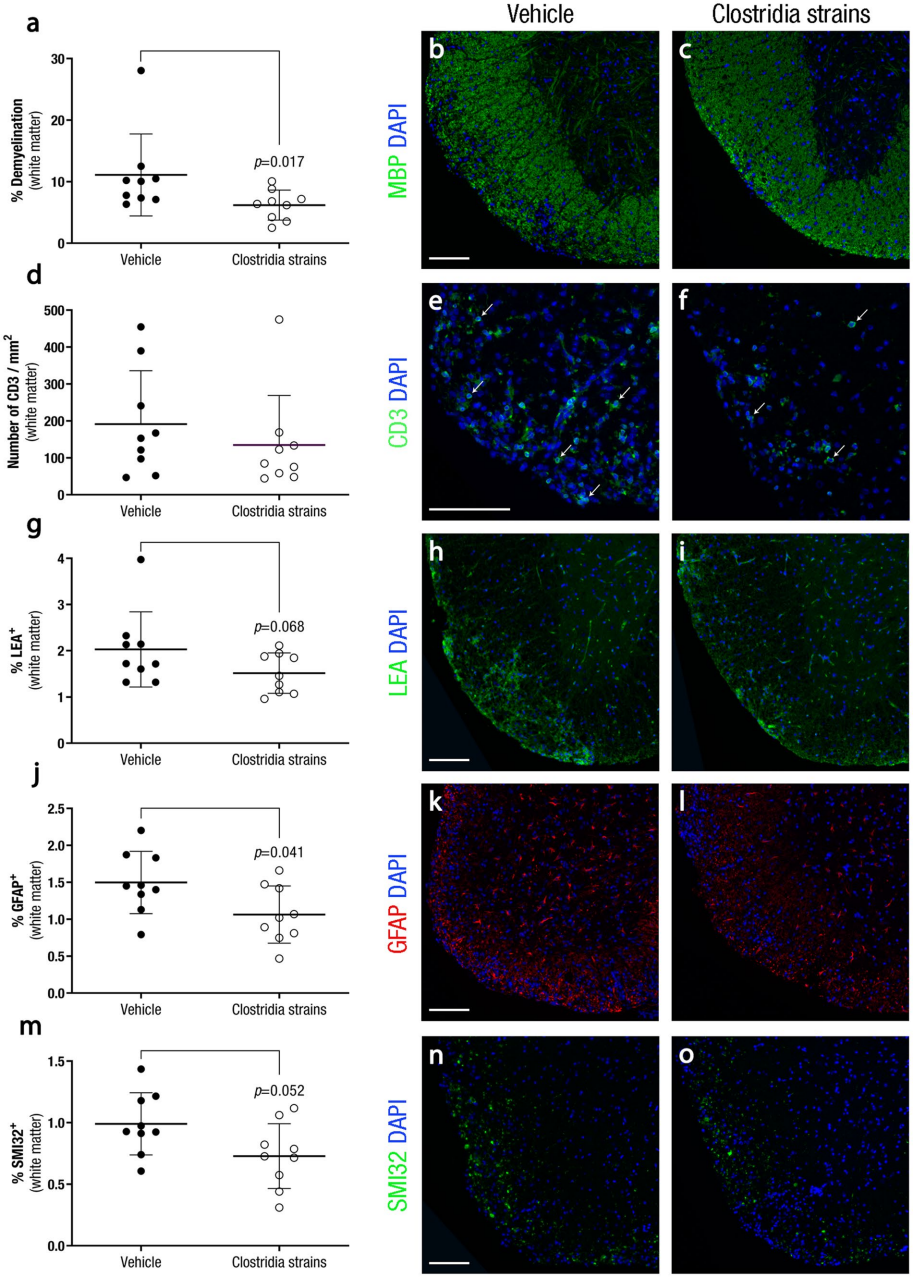
**Clostridia Strains Ameliorate Histopathological signs in the CNS of EAE Mice.** At 28 dpi, spinal cords of euthanised EAE mice were collected, fixed in a 4% paraformaldehyde solution, embedded in paraffin, and cut into 4-μm thick coronal sections. Demyelination was assessed by anti-myelin basic protein (MBP) antibody; T cell infiltrates, by anti-CD3 antibody; reactive microglia/infiltrating macrophages and astroglia reactivity, by lectin from *Lycopersicon esculentum* and anti-glial fibrillary acidic protein (GFAP) antibody, respectively; and axonal damage, by anti-neurofilament H, nonphosphorylated (SMI32). Daily therapeutic administration of Clostridia strains reduces demyelination **a**-**c** and reactive astroglia **j**-**l**, does not modify T cell infiltrates **d**-**f** and tends to diminish reactive microglia/infiltrating macrophages **g**-**i** and axonal damage **m**–**o**. The graphs present the results of a representative experiment (n = 9 mice per group). Arrows indicate CD3^+^ cells. Scale bar indicate 100 µm. The data are presented as the means ± standard deviations. Abbreviations: DAPI: 4′,6-diamidino-2-phenylindole, GFAP: glial fibrillary acidic protein, LEA: lectin from *Lycopersicon esculentum*, MBP: myelin basic protein, SMI32: neurofilament H, nonphosphorylated

Within the spinal cord white matter, demyelination was lower in mice treated with Clostridia strains (6·19 ± 2·44%, n = 9, *p* = 0·017) than in vehicle-treated mice (11·10 ± 6·66%, n = 9) (Fig. 2a-c). CNS inflammation was evaluated measuring T cell inflammatory infiltrate density and reactive microglia and infiltrating macrophages presence. No differences were observed in T cell inflammatory infiltrate (*p* = 0·364) (Fig. 2d-f) but a tendency to lower reactive microglia and infiltrating macrophages was observed in the Clostridia-treated group (1·52 ± 0·43%, n = 9, *p* = 0·068) compared to vehicle-treated mice (2·03 ± 0·81%, n = 9) (Fig. 2g-i). Regarding astrocyte reactivity, a reduction was observed in the Clostridia-treated mice (1·06 ± 0·39%, n = 9, *p* = 0·041) compared to the vehicle group (1·50 ± 0·42%, n = 9) (Fig. 2j-l). Finally, the level of axonal damage was also lower in mice treated with the Clostridia strains (0·73 ± 0·26%, *p* = 0·052) than in the vehicle group (0·99 ± 0·25%, n = 9), although no statistical significance was reached (Fig. 2m-o).

### Clostridia Strains Increase Antigen-Specific Proliferation but do not Alter Disease-Related Cytokines

**Fig. 3:**
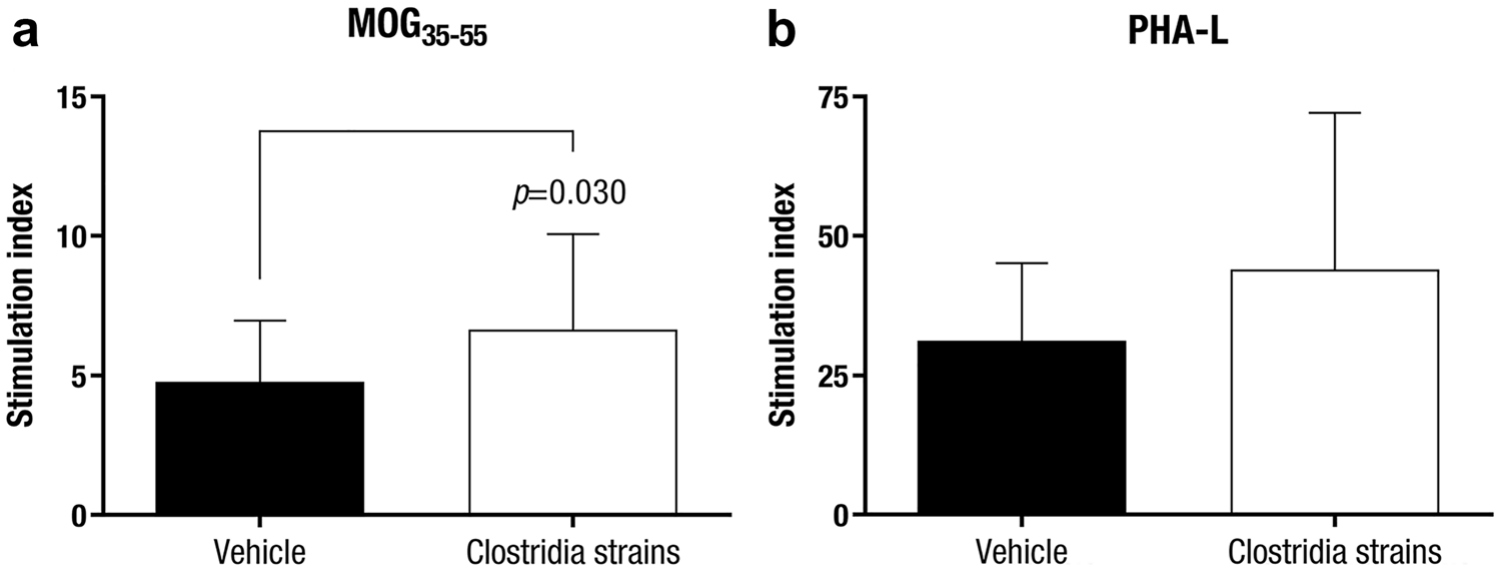
**Clostridia Strains Increase Autoreactive Splenocytes*ex vivo.*** Splenocyte cultures were prepared at the end of the experiment (28 dpi) and stimulated with 5 μg/ml MOG_35-55_ or 5 μg/ml PHA-L and compared to non-stimulated (control) condition. After 54 h in vitro, supernatants were harvested to further assess cytokine secretion and cell cultures were supplemented with [^3^H]-thymidine to quantify cell proliferation at 72 h *in vitro*. Clostridia strains increase the proliferative capacity of antigen-specific cells in response to MOG stimulation **a** but do not alter the polyclonal response **b**. The graphs present the combined results of two independent experiments (n = 15 mice per group). The data are presented as the means ± standard deviations. Abbreviations: MOG_35-55_: peptide 35–55 from myelin oligodendrocyte glycoprotein, PHA-L: phytohaemagglutinin-L

Whereas an increase in antigen-specific proliferation was observed under Clostridia strains treatment (stimulation index: 6·66 ± 3·41, n = 15, *p* = 0·030) compared to the vehicle group (stimulation index: 4·78 ± 2·21, n = 13) (Fig. 3a), no differences were detected in the polyclonal immune response (Fig. 3b). In addition, the cytokine secretion pattern in the supernatants of the antigen-specific stimulated splenocytes did not differ between these two experimental groups and some of them (IL-12p70, IL-21 and IL-23) were even not detected (Supp. Figure 1). Likewise, when we specifically measured the intracellular cytokine (IFN-γ, IL-4, IL-10, and IL-17A) production patterns in T helper (Th) and cytotoxic T cells from the periphery, no differences were found (*data not shown*).

### Clostridia Strains Increase the Immunoregulatory Capacity of Treg Cells

**Fig. 4:**
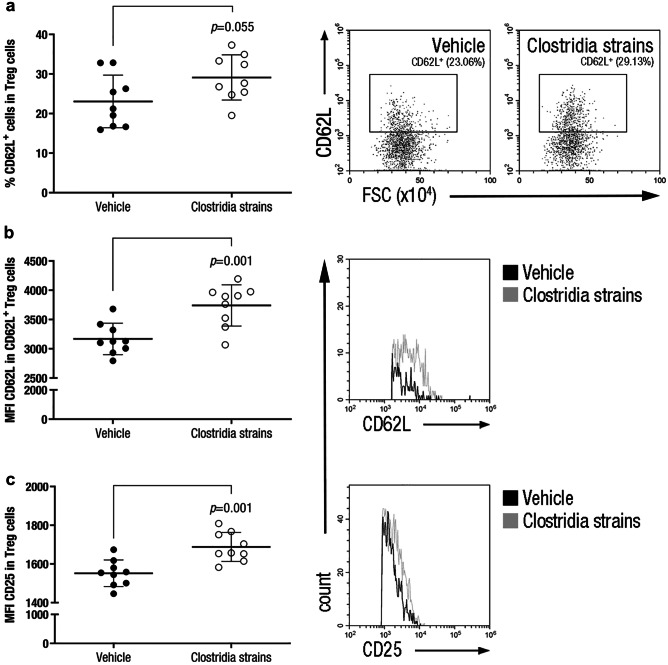
**Clostridia Strains Increase Immunoregulatory Properties of Treg Cells in the Periphery.** Splenocyte suspensions were prepared by grinding spleens of EAE mice through a 70-μm nylon cell strainer at 28 dpi. Cell subsets were analysed after exclusion of doublets and dead cells from the gating scheme. Clostridia treatment increases the CD62L^+^ Treg cell population **a** and the expression of CD62L **b** and CD25 **c** inducible surface markers in Treg cells in the periphery. Representative dot plots **a** or histograms **b**, **c** of every inducible surface marker in Treg cells are shown. The graphs present the results of a representative experiment (n = 9 mice per group). The data are presented as the means ± standard deviations. Abbreviations: MFI: median fluorescence intensity.

Immunoregulatory responses are implicated in the resolution of inflammation and can potentially alleviate clinical signs in the EAE model. In this sense, the increase in the CD62L^+^ Treg cell population in mice treated with Clostridia strains (29·13 ± 5·73%, n = 9, *p* = 0·055) compared to vehicle-treated mice (23·06 ± 6·66%, n = 9) could be related to the observed clinical improvement (Fig. 4a), although no statistical significance was reached. Moreover, the expression of the inducible molecules CD62L and CD25 was augmented in Treg cells of mice treated with Clostridia strains (CD62L: 3741·50 ± 353·03 MFI, n = 9, *p* = 0·001; and CD25: 1688·18 ± 75·23 MFI, n = 9, *p* = 0·001) compared to that in the vehicle group (CD62L: 3169·08 ± 268·67 MFI, n = 9; and CD25: 1551·92 ± 68·57 MFI, n = 9) (Fig. 4b,c). No differences were observed between the experimental groups in the rest of the studied Treg cell subsets (*data not shown*).

**Fig. 5:**
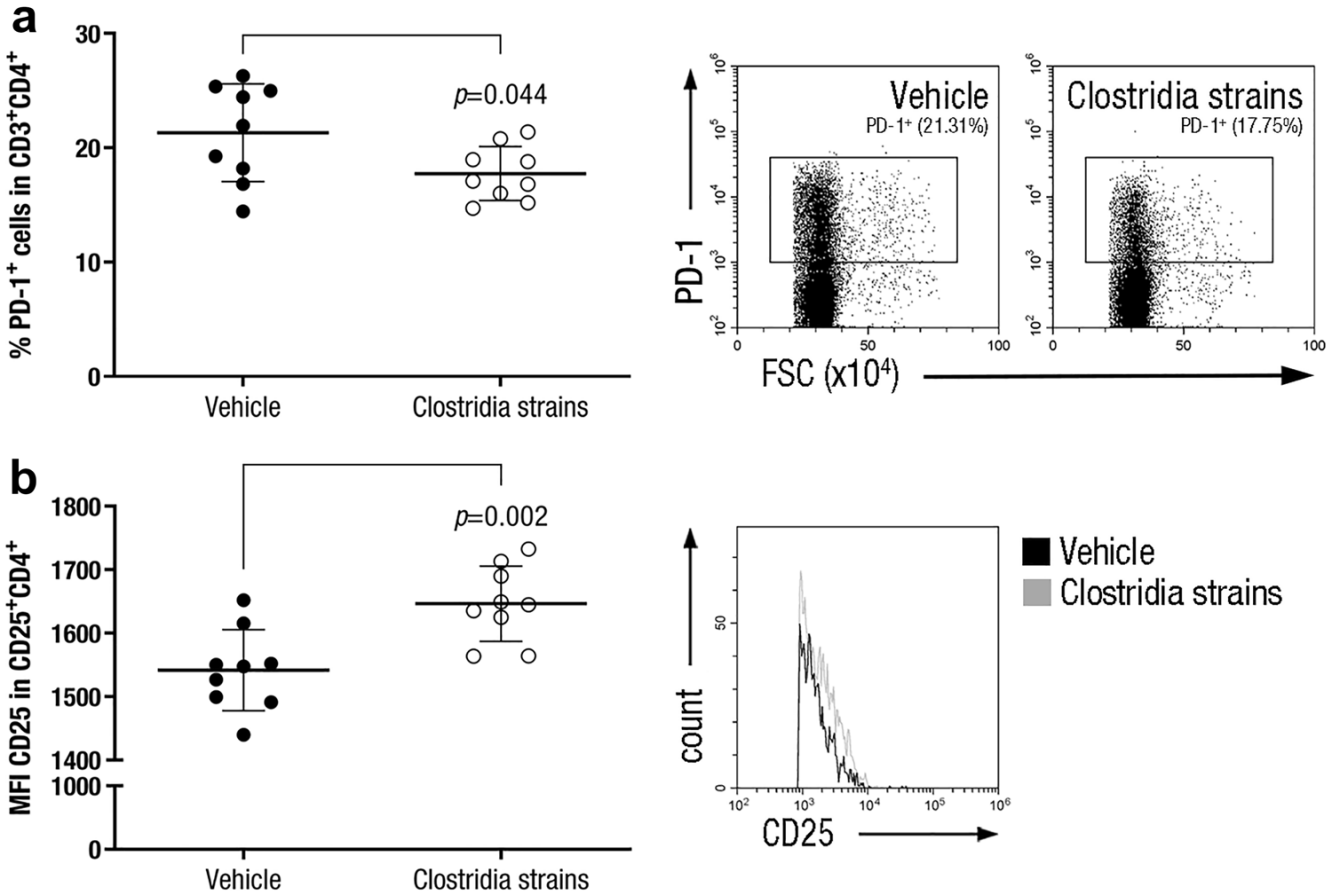
**Clostridia Strains Modify Th Population in the Periphery.** Splenocyte suspensions were prepared by grinding spleens of EAE mice through a 70-μm nylon cell strainer at 28 dpi. Cell subsets were analysed after exclusion of doublets and dead cells from the gating scheme. Clostridia-treated mice presented a decrease in the inducible PD-1^+^ Th cell population **a** and increased levels of CD25 activation marker in CD4^+^ T lymphocytes **b**. Representative dot plots **a** and histogram **b** are shown. The graphs present the results of a representative experiment (n = 9 mice per group). The data are presented as the means ± standard deviations. Abbreviations: MFI: median fluorescence intensity

On the other hand, Clostridia-treated mice presented a decrease in the PD-1^+^ Th cell population (17·75 ± 2·37%, n = 9, *p* = 0·044) compared to vehicle-treated mice (21·31 ± 4·27%, n = 9) (Fig. 5a), although no statistically significant differences were observed regarding PD-1 expression level (*data not shown*). Further study of the T cell activation status (CTLA-4, LAG-3, and TIM-3) did not show differences between experimental groups (*data not shown*). Moreover, Clostridia-treated mice presented increased levels of CD25 activation marker in CD4^+^ T lymphocytes (1646·43 ± 59·14 MFI, n = 9, *p* = 0·002) compared with vehicle-treated animals (1541·57 ± 64·04 MFI, n = 9) in the periphery (Fig. 5b). No differences were observed between experimental groups regarding B cell and myeloid cell [dendritic cells (DCs), macrophages, neutrophils, and myeloid-derived suppressor cells (MDSCs)] populations (*data not shown*).

### Clostridia Strains Reduce Immunological Responses in the CNS and Promote an IFN-β Response in the Periphery

Prior to studying the differentially expressed genes in our target tissues: *spinal cord* and *splenocytes*, different types of quality controls were performed to confirm that every array was suitable for normalisation process and, once normalised, that it was adequate for differential expression analysis. Later, a batch factor was included in the linear model used for differential expression analysis when required. In order to increase statistical power and reduce unnecessary noise, those genes whose standard deviation was below the 65 percentile of all standard deviations were removed. A list of 7,104 genes was included in the differential expression analysis. After correcting for multiple testing by the Benjamini and Hochberg method [20], no genes were found differentially expressed in Clostridia-treated mice compared to vehicle mice in either of our target tissues with an *adjusted p value* of 0·25 or below. Thus, no individual gene met the threshold for statistical significance possibly because the relevant biological differences are modest in relation to the noise of the microarray technology. In order to overcome this setback, we performed a gene set enrichment analysis (GSEA) to determine whether members of a given *Gene Set* correlate with the phenotypic class distinction between our experimental groups (Clostridia- and vehicle-treated mice) within the list of selected genes (7,104 genes) [18].

**Fig. 6:**
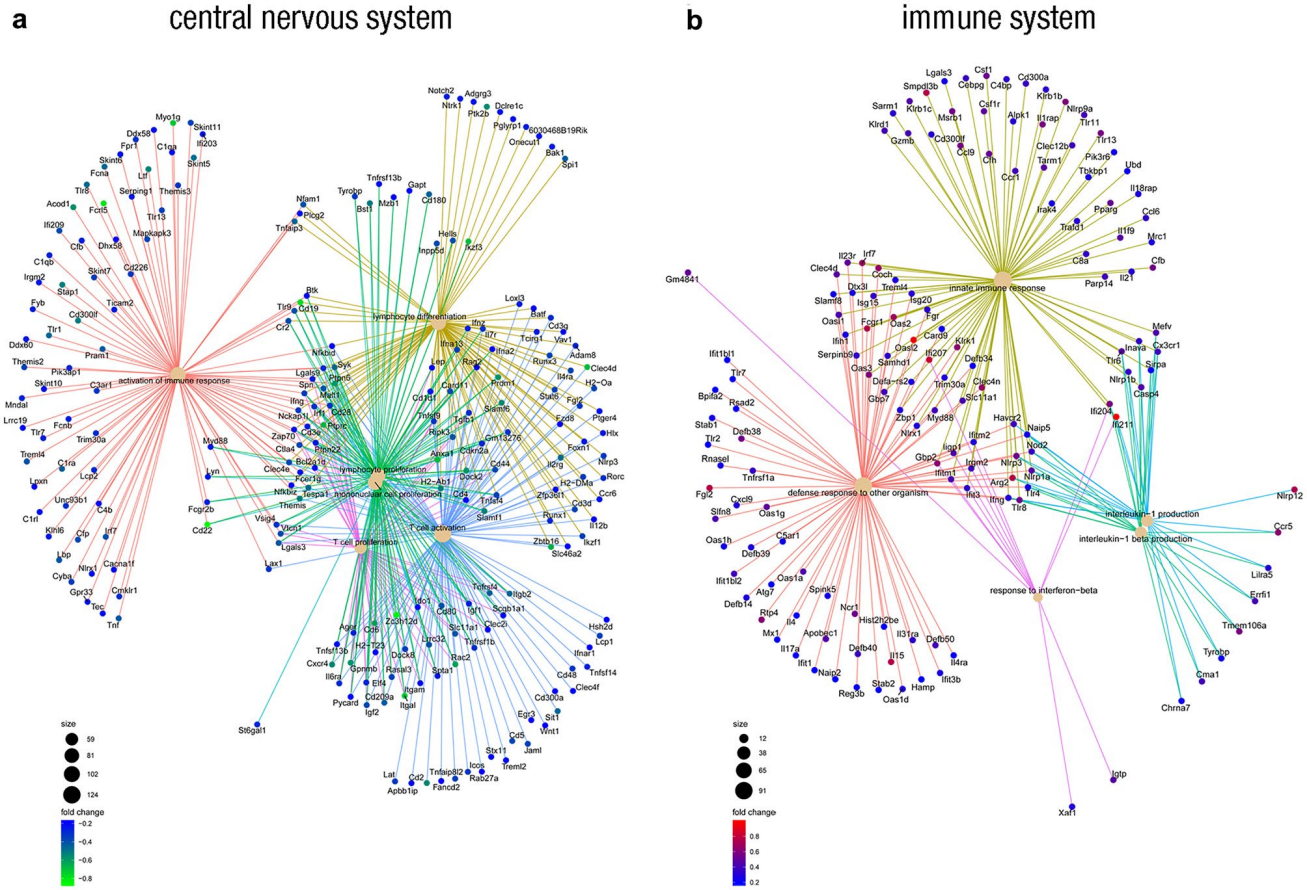
**Clostridia Strains Reduce the Immune Response in the CNS and Increase the Innate Immune Response in the Periphery.** At 28 dpi, spinal cords and splenocytes of euthanised mice were collected and total RNA was isolated. Subsequently, cDNA was generated and Clariom S Pico Assay HT, mouse arrays were processed. The analysis of biological significance, based on the GSEA method, highlights that Clostridia strains alter different gene sets from the "Gene Ontology" (GO) database in both the CNS **a** and the peripheral immune system **b**. Specifically, Clostridia strains reduced: 1) the activation of the immune response and T cells, in particular; 2) the differentiation of lymphocytes; and 3) the proliferation of mononuclear cells and lymphocytes and T cells, specially, in the CNS. On the other hand, Clostridia strains promote gene sets related to: 1) the defense response to other organisms; 2) the innate immune response; and 3) IL-1, IL-1β, and IFN-β production. In these plots, the *size* of the GO term nodes is related to the number of genes found in that category, the *color of the gene nodes* refers to their logFC, and the *color of the edges* links each gene with its GO term

Clostridia strains reduced the activation of the immune response in general and T cells in particular, the lymphocyte differentiation, and the mononuclear cell proliferation (T cells and lymphocytes, specially) in the CNS of EAE mice [*adjusted p* < 0·05 and normalised enrichment score (NES) < -2·3] (Fig. 6a). Besides these biological processes (GO database), Clostridia treatment was also related to the reduction of several pathways (Reactome Pathway Knowledge base) related to the immune response: immunoregulatory interactions between lymphoid and non-lymphoid cells, cell surface interactions at the vascular wall, innate immune system, and IL-2 family signaling (*adjusted p* < 0·05 and NES < -2). Regarding the biological effect of Clostridia treatment in the periphery, Clostridia-treated mice showed an increased defence response to other organisms that was partially related to the innate immune response, the IL-1 production, and the response to IFN-β (*adjusted p* < 0·1 and NES > 2) (Fig. 6b). This defence response was probably related to the high number of microorganisms administered to mice and it was in line with the immune pathways observed: neutrophil degranulation and innate immune system (*adjusted p* < 0·05 and NES > 1·5).

### Clostridia Strains Increase the Levels of the SCFA Butyrate in Mouse Serum

Since the administered Clostridia strains are highly known for their capacity of SCFA production, we studied whether the levels of several SCFA such as acetate, propionate, and butyrate, were altered as a result of the oral administration of these bacteria. The concentration of the SCFA butyrate was increased in the serum of Clostridia-treated mice (1·74 ± 0·40 µM, n = 6, *p* = 0·047) compared to that in vehicle-treated mice (1·23 ± 0·33 µM, n = 5) (Fig. 1f) but no differences were observed in the rest of the studied SCFAs (*data not shown*).

### Butyrate Ameliorates the Clinical Signs and Reduces CNS Autoimmunity in EAE Mice

Since Clostridia strains increased the production of the immunoregulatory SCFA butyrate, we studied whether the previously observed clinical effect could be reproduced by butyrate administration alone.

**Fig. 7:**
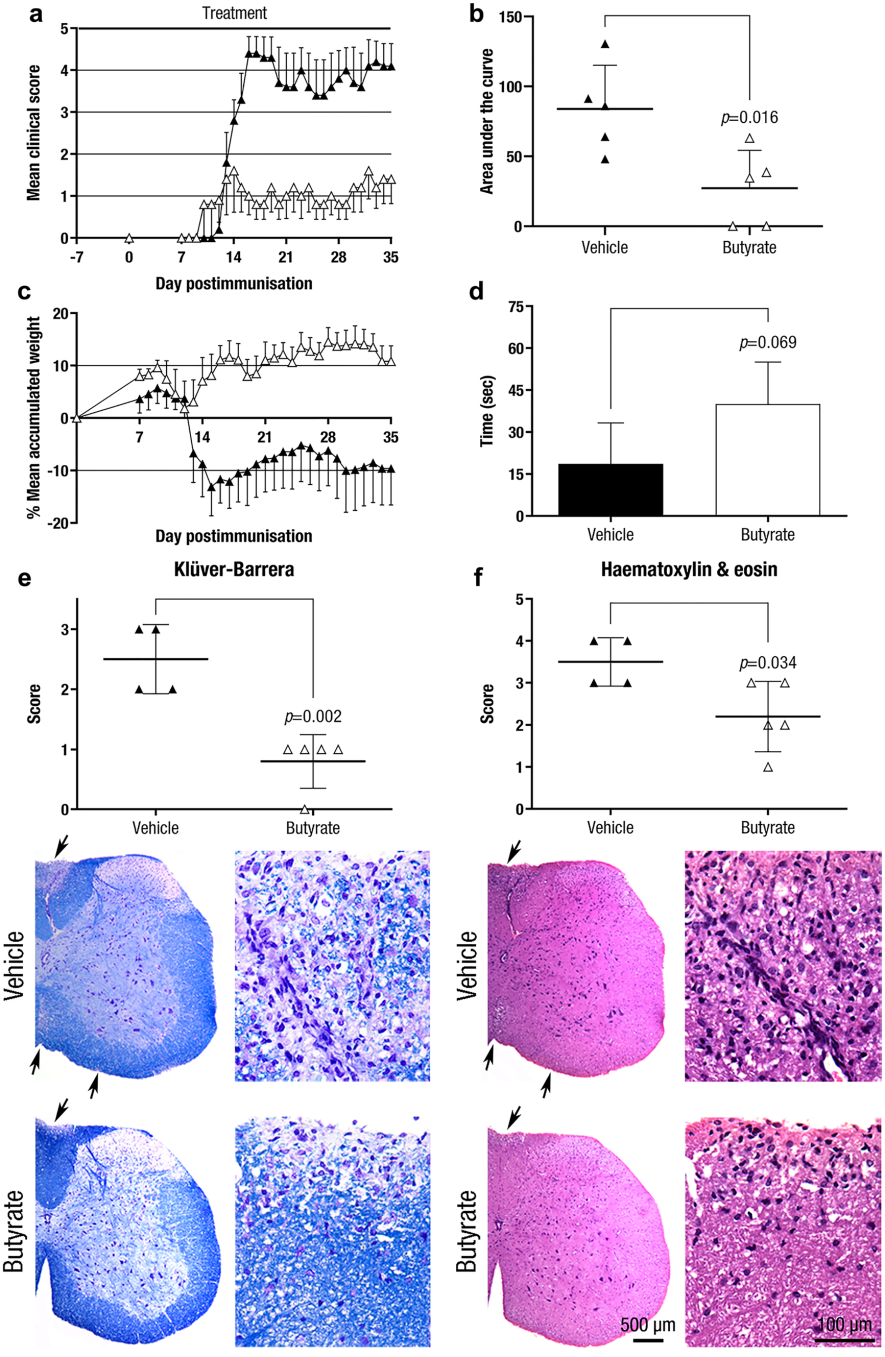
**Butyrate Treatment Reduces the Pathological Clinical Signs and the CNS Inflammation and Demyelination of EAE Mice as a Preventive Approach.** Daily oral preventive treatment with sodium butyrate (daily intake: 90·67 ± 19·38 mg) from 7 days before mice immunisation until the end of the experiment (35 dpi) improves clinical score **a**, AUC **b**, weight loss **c**, and tends to improve motor function skills **d** compared to vehicle group. Moreover, sodium butyrate reduces CNS demyelination **e** and inflammation **f**. The graphs present the results of a single experiment (n = 5 mice per group). Arrows indicate demyelinated and inflamed areas within the spinal cord white matter. Scale bars indicate 100 µm or 500 µm. The data are presented as the means ± standard errors of the mean **a**, **c** or the means ± standard deviations **b**, **d**-**f**. Black triangle, Vehicle; white triangle, Butyrate

Daily intake of butyrate (90·67 ± 19·38 mg/mouse), from seven days before mice immunisation to the end of the experiment (35 dpi), significantly reduced the clinical signs of EAE mice (AUC: 27·20 ± 27·12, n = 5, *p* = 0·016) compared to vehicle treatment (AUC: 83·95 ± 31·28, n = 5) as a preventive approach (Fig. 7a,b). Butyrate intake not only affected clinical score but also modified the maximum score (2·20 ± 2·05, n = 5, *p* = 0·049) compared to vehicle treatment (4·50 ± 0·87, n = 5). As expected, butyrate-treated mice also reflected their clinical improvement in other clinical variables such as the accumulated weight loss (AUC: 318·30 ± 187·50, n = 5, *p* = 0·033) and motor coordination skills (40·02 ± 15·00 s, n = 5, *p* = 0·069) compared to vehicle group (AUC: -152·90 ± 364·50, n = 5; and motor coordination skills: 18·65 ± 14·60 s, n = 4) (Fig. 7c,d).

Within the spinal cord white matter, histopathological signs such as demyelination (demyelination score: 0·80 ± 0·45, n = 5, *p* = 0·002) (Fig. 7e) and CNS inflammation (cell inflammation score: 2·20 ± 0·84, n = 5, *p* = 0·034) (Fig. 7f) were diminished in the butyrate-treated mice compared to the vehicle group (demyelination score: 2·50 ± 0·58, n = 4; and cell infiltration score: 3·50 ± 0·58, n = 4).

On the other hand, antigen-specific (MOG_35–55_) and polyclonal (PHA-L) immune responses were not altered by the preventive administration of the SCFA butyrate (stimulation index: 4·62 ± 2·32, n = 5, *p* = 0·394 and 31·43 ± 8·05, n = 5, *p* = 0·894, respectively) compared with those in the vehicle group (stimulation index: 3·61 ± 0·56, n = 4 and 32·30 ± 10·86, n = 4, respectively) (*data not shown*). Finally, no between-group differences in the cytokine secretion pattern (GM-CSF, IFN-γ, IL-2, IL-4, IL-6, IL-10, IL-12p70, IL-17A, IL-21, IL-22, IL-23, and TNF-α) were observed for antigen-specific stimulatory condition (*data not shown*).

**Fig. 8:**
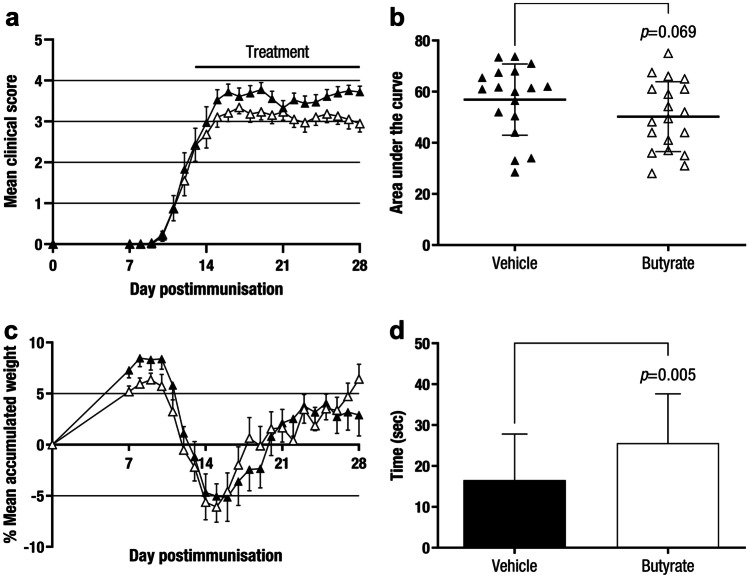
**Therapeutic Administration of Butyrate Causes Slight Clinical Effects in EAE Mice.** C57BL/6JOlaHsd mice were immunised by subcutaneous injection of MOG_35–55_ and weighed and examined daily for neurological signs. After attaining a clinical score equal to or greater than 1 and being randomised (13–15 dpi), mice were treated once daily with sodium butyrate (100 mg/dose) or water (vehicle), weighed and examined daily for neurological signs in a blinded manner until the end of the experiment (28 dpi). Daily therapeutic administration of butyrate slightly improves the clinical score **a** and the AUC **b** and does not affect weight loss **c**. Furthermore, butyrate improves motor function skills compared to vehicle group **d**. The charts present the combined results of three independent experiments (Vehicle, n = 18 and Butyrate, n = 19). The data are presented as the means ± standard errors of the mean **a**, **c** or the means ± standard deviations **b**, **d**. Black triangle, Vehicle; white triangle, Butyrate

Next, we tested the therapeutic effect of butyrate administration as a potential translational approach. Since a mean daily butyrate intake (90·67 ± 19·38 mg/mouse) was estimated in the preventive approach and it was identified as an effective dose in the EAE model, 100 mg of this SCFA was selected as the suitable butyrate quantity for the therapeutic approaches. Mice were treated once daily with butyrate or vehicle after attaining a clinical score equal to or greater than 1 and being randomised into clinically equivalent experimental groups, from 13–15 dpi to the end of the experiment (28 dpi). A slight clinical effect was observed in the butyrate group (AUC: 50·24 ± 13·64, n = 19, *p* = 0·069) compared to that in vehicle-treated mice (AUC: 56·89 ± 13·93, n = 18) (Fig. 8a,b), although these differences were not statistically significant. No differences were observed regarding accumulated weight loss (Fig. 8c) but, compared to the vehicle group (16·57 ± 11·23 s, n = 17), butyrate treatment improved motor coordination skills (25·61 ± 12·05 s, n = 19, *p* = 0·005) (Fig. 8d).

**Fig. 9:**
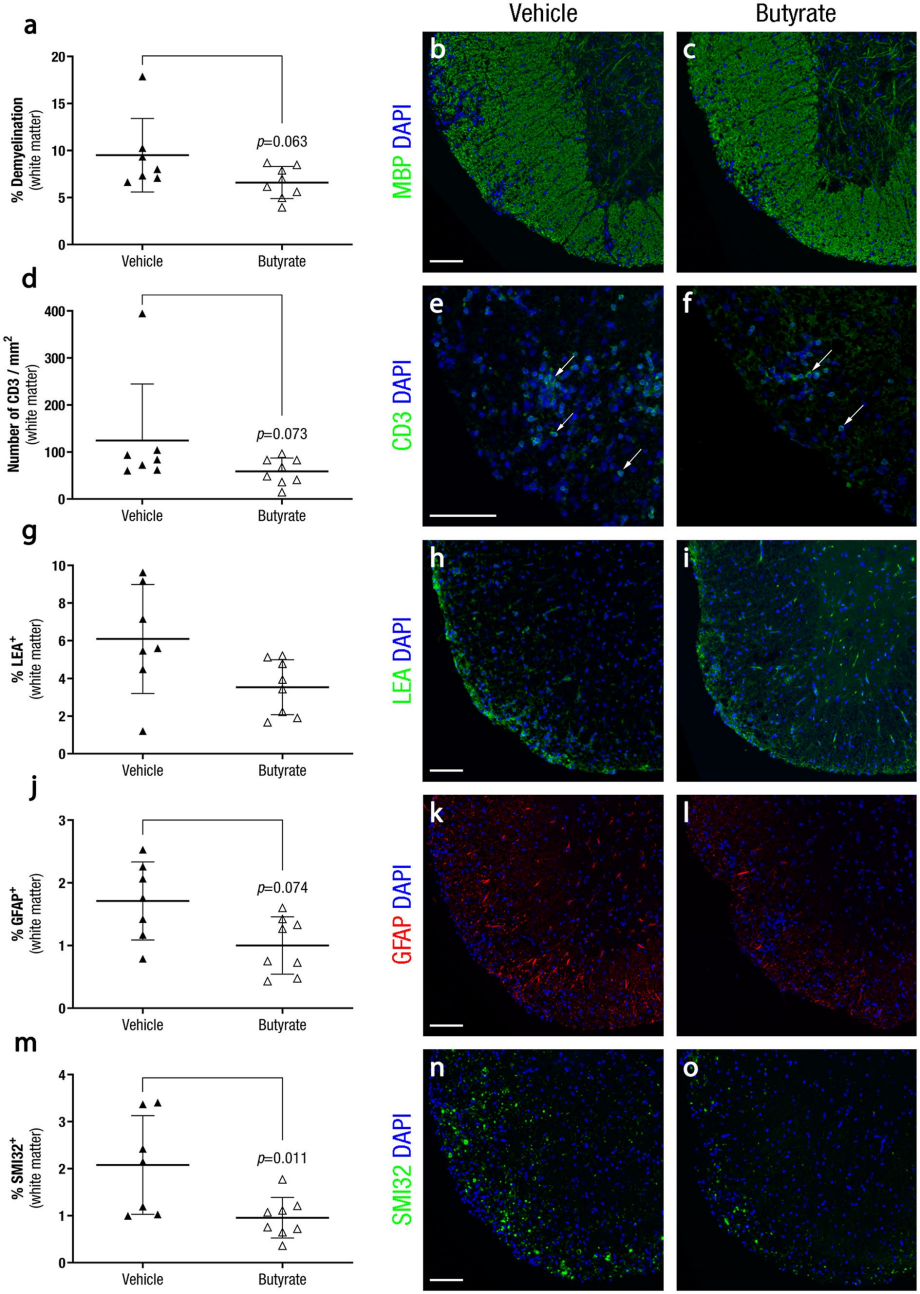
**Some Pathological Signs are Improved by the Therapeutic Administration of Butyrate in EAE Mice.** At 28 dpi, spinal cords of euthanised EAE mice were collected, fixed in a 4% paraformaldehyde solution, embedded in paraffin, and cut into 4-μm thick coronal sections. Demyelination was assessed by anti-myelin basic protein (MBP) antibody; T cell infiltrates, by anti-CD3 antibody; microglia reactivity/infiltrating macrophages and reactive astroglia, by lectin from Lycopersicon esculentum and anti-glial fibrillary acidic protein (GFAP) antibody, respectively; and axonal damage, by anti-neurofilament H, nonphosphorylated (SMI32). Therapeutic administration of butyrate decreases axonal damage **m**–**o**, tends to reduce CNS demyelination **a**-**c**, T cell infiltration **d**-**f** and reactive astroglia **j**-**l**, and does not alter microglia reactivity/infiltrating macrophages **g**-**i** in the spinal cord white matter. The graphs present the results of a representative experiment (Vehicle, n = 7; and Butyrate, n = 8). Arrows indicate CD3^+^ cells. Scale bars indicate 100 µm. The data are presented as the means ± standard deviations. Abbreviations: DAPI: 4′,6-diamidino-2-phenylindole, GFAP: glial fibrillary acidic protein, LEA: lectin from *Lycopersicon esculentum*, MBP: myelin basic protein, SMI32: neurofilament H, nonphosphorylated

Within the spinal cord white matter, demyelination (6·59 ± 1·71%, n = 8, *p* = 0·063), T cell inflammatory infiltrate density (58·83 ± 28·18 cells/mm^2^, n = 8, *p* = 0·073), and astroglia reactivity (1·00 ± 0·46%, n = 8, *p* = 0·074) were slightly reduced in butyrate-treated mice compared to vehicle treatment (demyelination: 9·50 ± 3·91%, n = 7; T cell inflammatory infiltrate density: 124·42 ± 120·38 cells/mm^2^, n = 7; and astroglia reactivity: 1·71 ± 0·62%, n = 7) (Fig. 9a-c, 9d-f, and 9j-l), although differences did not reach statistical significance. Regarding microglia reactivity and infiltrating macrophages, no statistically significant differences were observed between the butyrate (3·54 ± 1·46%, n = 8, *p* = 0·127) and the vehicle group (6·10 ± 2·89%, n = 7) (Fig. 9g-i). However, the level of axonal damage was lower in mice treated with butyrate (0·96 ± 0·43%, n = 8, *p* = 0·011) than in the control group (2·08 ± 1·05%, n = 7) (Fig. 9m-o). As seen in the preventive approach, butyrate treatment did not modify antigen-specific (stimulation index: 6·52 ± 3·33, n = 11 vs. 7·75 ± 2·74, n = 10, *p* = 0·129, respectively) or polyclonal immune responses (stimulation index: 28·41 ± 24·27, n = 11 vs 26·28 ± 10·83, n = 10, *p* = 0·577, respectively) compared to the vehicle group as a therapeutic approach (*data not shown*).

## Discussion

Our results show that oral treatment with human gut-derived Clostridia strains improves the clinical outcome of established EAE as a therapeutic approach. Previous studies showed that these 17 Clostridia strains performed both clinical and histological benefits in an experimental model of colitis [10]. These bacteria were originally isolated from a healthy human faecal sample and their selection was grounded in their capacity of inducing Treg cells [10]. As the selected bacteria belong to Clostridia clusters IV, XIVa, and XVIII and it has been reported that MS patients present a significant decrease of Clostridia clusters IV and XIVa [5], we thought that not only this bacteria cluster could increase immunoregulatory responses but also it could have the potential to partially rebalance gut dysbiosis in MS patients. Our study is one of the few preclinical assays that uses human isolated bacteria in an EAE model [21, 22]. Likewise, it is the first one that provides commensal bacteria with the potential to partially rebalance gut dysbiosis in MS patients.

Previous studies in EAE and MS patients have observed alterations in the intestinal permeability [16, 23, 24]. It has been proposed that this intestinal dysfunction could be contributing to disease progression or the pathophysiology of the disease. In fact, intestinal permeability increases as early as in the induction and the inflammatory phases of EAE and CNS autoreactive T cells have been related to this damaged intestinal integrity [16, 24]. However, no differences in gut permeability were observed between our experimental groups when intestinal permeability was evaluated in the chronic phase of the experimental disease. Conversely, other studies have revealed a reduced intestinal permeability in probiotic-treated mice in both the induction and the inflammatory phases of EAE as a preventive approach [24]. Keeping this in mind, it is possible that once intestinal features are established, the ability to revert this intestinal dysfunction may be limited.

We observed a decrease of CNS demyelination in accordance to clinical features in Clostridia-treated group. Moreover, we wanted to deepen into other important pathological hallmarks of the experimental disease, such as T cell infiltration, reactive microglia/infiltrating macrophages and astrocyte reactivity, and axonal damage. Although every pathological feature tended to decrease in line with the attenuation of clinical signs, only astrogliosis was significantly affected by Clostridia strains administration.

Regarding the immunological effects, Clostridia strains only performed subtle immune regulation in the periphery. Whereas a higher antigen-specific immune proliferation was detected under Clostridia treatment, which was initially thought to be adverse, this immune response did not have a negative effect on the CNS pathology, the cytokine secretion pattern from MOG-stimulated immune cells, or any other studied immune response in the periphery. On the contrary, this result could be in line with the observed increase in the CD62L^high^ Treg cell subset in the periphery. However, since immunophenotyping was not performed within the antigen-specific cells, we cannot explain the increased immune cell proliferation by means of this Treg cell population alone.

In the transcriptome studies, an activation of the innate immune system was observed under Clostridia treatment. We hypothesise that, rather than a disease-related immune response, the high number of administered bacteria could be promoting this innate immune response in the periphery. On the other hand, transcriptome studies also highlighted an upregulation of genes related to the response to IFN-β in the periphery of Clostridia-treated mice. Whereas this cytokine could also be fostered as a response to bacterial infection, the biological or chemical stimuli that triggered this cytokine by the immune system have not been studied. It is relevant to mention that IFN-β is a first-line drug to treat MS and it has been described to ameliorate EAE clinical course. Moreover, the deletion of the IFN-β gene has been shown to worsen EAE clinical course [25]. Regarding its mechanisms of action, some studies have demonstrated that its antiinflammatory effect is mediated by the innate immune cells that inhibit proinflammatory Th17 cells [26, 27], whereas other groups have described that IFN-β would be inhibiting T cell activation, reducing immune cell trafficking across the BBB, or inducing antiinflammatory cytokines among others [28]. Together with the enhanced response to IFN-β, an increase in the genes related to the production of IL-1β in the periphery was also observed in Clostridia-treated mice. This result may be in line with previous studies in EAE and MS, which pointed to a role of the NLRP3 inflammasome and its related cytokine, IL-1β, in the effective clinical response to IFN-β in EAE mice and RRMS patients [29, 30].

Although Clostridia strains treatment did not modify the overall Treg cell population in the periphery, it increased both the percentage of Treg cells that express CD62L and the expression level of this surface molecule within Treg cell population. It has been previously described that CD62L^high^ Treg cells displayed higher immunosuppressive properties due to a higher ability to suppress proliferation of effector T cells and to induce apoptosis in Th cells *in vitro* [31]. Clostridia treatment also promoted higher CD25 (IL-2 receptor α-chain) levels in the cell surface of Treg and Th cells compared to vehicle treatment, these latter depending directly on the former. High levels of IL-2 receptor in Treg cells have been proposed as an immunoregulatory mechanism that deprives proliferating effector cells from this growth factor [32, 33]. Consequently, a lower availability of IL-2 will diminish effector immune cell proliferation. Besides preventing proliferative processes, IL-2 deprivation also leaves effector cells without a key cytokine for metabolic processes and survival. Finally, Clostridia treatment presented a lower population of Th cells that express the immunoinhibitory receptor PD-1, which has been defined as an inducible molecule involved in the termination of the immune response after T cell activation [34]. This later effect suggests that a higher number of T cells from vehicle-treated mice have been previously activated and, therefore, a higher amount of Th cells expressed PD-1 molecule to negatively regulate excessive immune activation.

Within the CNS of Clostridia-treated mice, transcriptome studies highlighted the reduction in several biological processes related to the activation, differentiation, and proliferation of immune cells. Particularly, the reduction of the mononuclear cell proliferation was strongly connected to lymphocyte and T cell proliferation. Whereas the histopathological studies did not show a decrease in T cell density within the spinal cord white matter, the trend towards a lower LEA-positive cells (infiltrating macrophages and reactive microglia) is consistent with these gene expression results. A few immune pathways were also downregulated in the CNS of Clostridia-treated mice. The reduction in the cell surface interactions at the vascular level may be related to the downregulation of genes related to the immune cell proliferation in the CNS as well as to the upregulation of genes related to the response to IFN-β in the periphery. Since the IFN-β limits immune cell trafficking to the CNS by downregulating T cell adhesion molecules that interacts to BBB adhesion molecules [35], the lower cell surface interaction at the vascular level may be partially related to the diminished immune cell responses in the CNS. On the other hand, the reduction in the IL-2 signalling could also be associated to the downregulation of genes related to the immune cell proliferation in the CNS since IL-2 is the prototypical T cell growth factor. Finally, the decrease in the innate immune system pathway could partially be pointing to the reduction of macrophages in the CNS in line with the histopathological studies.

The SCFA butyrate and, to a lesser extent, propionate, have been demonstrated to foster *in vitro* activation of DCs and subsequent promotion of Treg cells as well as *in vivo* generation of Treg cells in peripheral compartments [11]. Moreover, SCFAs have been stood out as the real sources of the antiinflammatory immune responses exerted by some probiotic bacteria, including Clostridia strains [10]. Therefore, the increased levels of butyrate in the serum of Clostridia-treated mice indicated that this SCFA might be acting as an antiinflammatory molecule in the periphery. In fact, the presence of this bacterial product could partially explain the increase in Treg cell subsets as well as in their higher immunosuppressive properties. It is worth mentioning that the detection of butyrate in the mouse serum illustrates how the oral administration of these Clostridia strains promoted a systemic effect that links the gut environment with the peripheral immune compartment.

Since our results, together with previous studies, connected the SCFA butyrate with immunoregulatory responses, we decided to test the oral administration of butyrate in the chronic EAE model. In our experimental model, preventive administration of butyrate performed a beneficial effect on CNS autoimmunity by halting both demyelination and CNS inflammation. These results are in accordance with previous studies that reported a significant amelioration of CNS demyelination in a cuprizone model as a preventive approach [36]. Considering a potential translational approach, we tested the therapeutic effect of butyrate administration in the EAE model. A slight but not statistically significant clinical effect on established EAE was observed. A tendency to lower demyelination, CNS inflammation, and astrogliosis was detected in line with clinical outcome. On the contrary, a noticeable improve concerning axonal damage was observed. In this way, the remarkable difference on EAE clinical course between the preventive and the therapeutic approaches suggest that SCFA butyrate would be acting only on the development of the immune response and has little effect on the neuroinflammatory processes. However, the limited immune studies performed in this experimental model do not allow for understanding the mechanism of action of butyrate treatment on CNS autoimmunity. Our studies only proved that butyrate treatment does not modify proliferation or cytokine secretion pattern of antigen-specific cells *ex vivo*. Additionally, the SCFA butyrate seems to behave similar to propionate treatment since only preventive but not therapeutic oral administration after the EAE induction phase had a significant beneficial effect on EAE clinical course [12]. Nevertheless, the translational approach to evaluate propionate efficacy in MS patients did perform a beneficial clinical effect on MS disease course [37]. Oral propionate supplementation increased the number and immunoregulatory function of Treg cells and decreased the number of Th17 cells, reversing the Treg/Th17 cell imbalance in MS patients. Regarding the CNS compartment, oral propionate supplementation increased propionate concentration in the CSF and this, in turn, was associated to the increase in subcortical grey matter in treated MS patients. More importantly, MS patients who were continuously supplemented with SCFA propionate for at least one year reduced their annual relapse rate and disease progression [37]. Thus, this translational study made us aware of the therapeutic potential of butyrate administration even though its therapeutic clinical impact in the EAE model was not statistically significant. Further studies should be performed to investigate its immunoregulatory effect on EAE pathogenesis and to value its translational potential. On the other hand, the smaller therapeutic effect in the butyrate-treated animals than in Clostridia-treated mice, even though higher levels of the SCFA butyrate were present in the former group, highlighted that the beneficial outcome exerted by the oral administration of the bacteria was not only related to the production of the SCFA. Thus, the Clostridia-induced clinical effect could not be reproduced by butyrate administration alone.

Our data show that the human gut-derived Clostridia strains improved the clinical outcome of established EAE as a therapeutic approach. The observed clinical improvement was related to lower histopathological features in the CNS and was associated to an enhanced immunoregulatory response of Treg cells in the periphery. Likewise, transcriptome studies highlighted increased antiinflammatory responses related to IFN-β in the periphery and lower activation, differentiation, and proliferation of immune cells in the CNS. Further *in vivo* assays with the SCFA butyrate proved its preventive effect on CNS autoimmunity but a slight therapeutic clinical impact on EAE clinical course. Our results suggest that the therapeutic effect performed by this commensal bacteria population was not exclusively related to the SCFA and could not be reproduced by butyrate administration alone. Although it is still unknown whether these 17 Clostridia strains will have the same effect on MS patients, previously-defined gut dysbiosis regarding Clostridia cluster IV and XIVa in MS patients could be rebalanced by Clostridia strains administration and its immunoregulatory properties could have a beneficial effect on MS clinical course.

## Supplementary Information

Below is the link to the electronic supplementary material.

ESM 1 (PDF 1225 KB)

ESM 2 (PDF 1225 KB)

ESM 3 (PDF 1225 KB)

ESM 4 (PDF 1225 KB)

ESM 5 (PDF 1225 KB)

ESM 6 (PDF 1546 KB)

ESM 7 (PDF 1225 KB)

ESM 8 (PDF 1225 KB)

ESM 9 (PDF 1225 KB)

ESM 10 (PDF 1225 KB)

ESM 11 (PDF 1225 KB)

ESM 12 (PDF 1225 KB)

ESM 13 (PDF 1226 KB)

Supplementary file14 (TIF 24543 KB) Supp. Fig.1 Clostridia strains do not alter key disease-related cytokine secretion pattern in the supernatants of autoreactive splenocytes. Splenocyte cultures were prepared at the end of the experiment (28 dpi) and stimulated with 5 μg/ml MOG35-55. After 54 h *in vitro*, supernatants were harvested to further assess cytokine secretion pattern. Clostridia strains do not alter the secretion pattern of disease-related cytokines in the supernatants of autoreactive splenocytes. The graphs show the results of a representative experiment (Vehicle, n = 7; and Clostridia strains, n = 9). The data are presented as the means ± standard deviations. Abbreviations: GM-CSF: granulocyte–macrophage colony-stimulating factor, IFN: interferon, IL: interleukin, TNF-α: tumour necrosis factor alpha.
