# Supplementary figures and images for: Selected Clostridia Strains from The Human Microbiota and their Metabolite, Butyrate, Improve Experimental Autoimmune Encephalomyelitis
Source: Neurotherapeutics. 2021 Apr 7;18(2):920–37. doi: 10.1007/s13311-021-01016-7 (PMC8423884; doi:10.1007/s13311-021-01016-7)

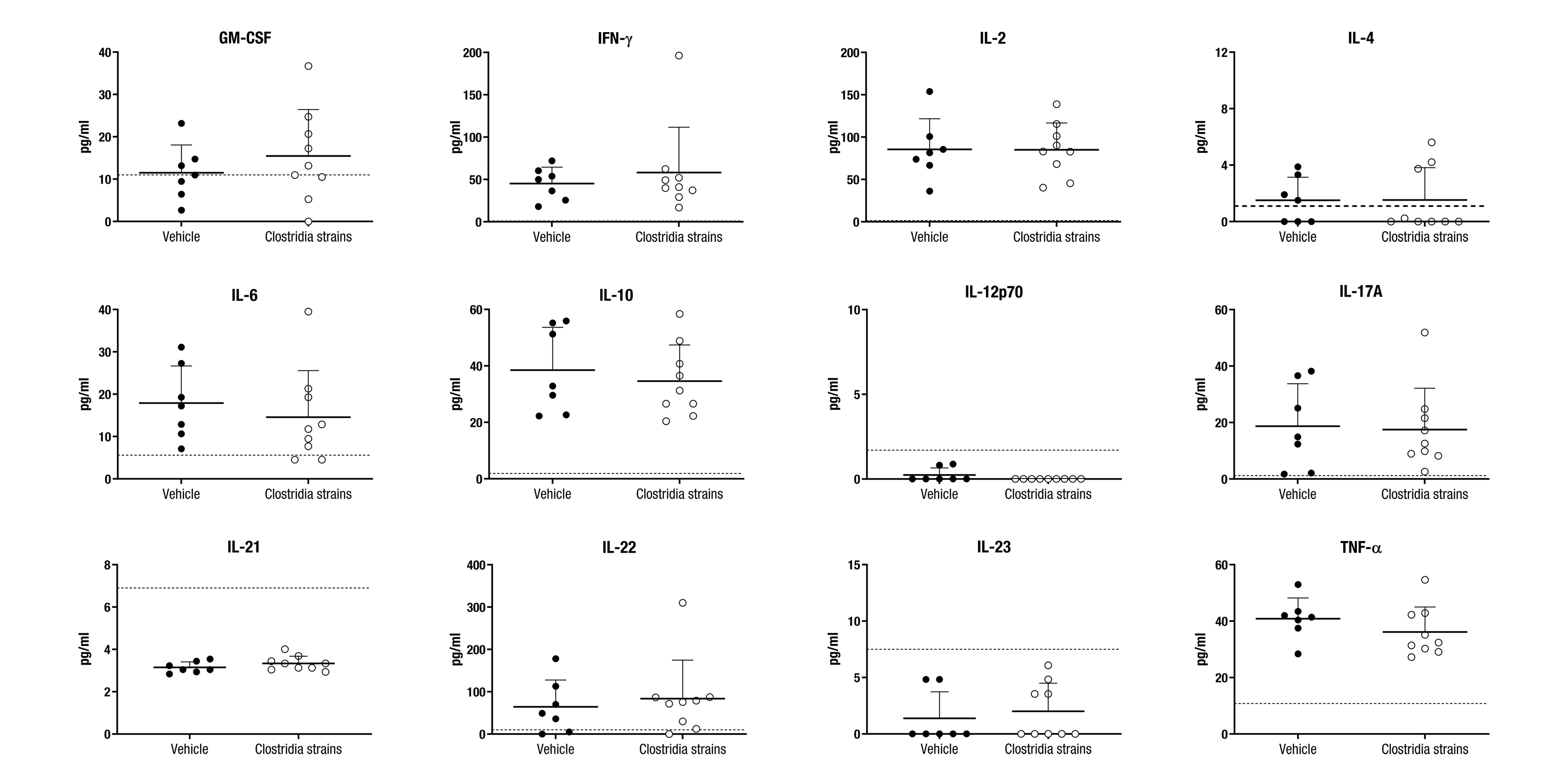

Supplement: Supplementary file 14 — Supplementary file14 (TIF 24543 KB) Supp. Fig.1 Clostridia strains do not alter key disease-related cytokine secretion pattern in the supernatants of autoreactive splenocytes. Splenocyte cultures were prepared at the end of the experiment (28 dpi) and stimulated with 5 μg/ml MOG35-55. After 54 h in vitro, supernatants were harvested to further assess cytokine secretion pattern. Clostridia strains do not alter the secretion pattern of disease-related cytokines in the supernatants of autoreactive splenocytes. The graphs show the results of a representative experiment (Vehicle, n = 7; and Clostridia strains, n = 9). The data are presented as the means ± standard deviations. Abbreviations: GM-CSF: granulocyte–macrophage colony-stimulating factor, IFN: interferon, IL: interleukin, TNF-α: tumour necrosis factor alpha. [file 13311_2021_1016_MOESM14_ESM.tif]
